# Portable Electronic Nose Based on Digital and Analog Chemical Sensors for 2,4,6-Trichloroanisole Discrimination

**DOI:** 10.3390/s22093453

**Published:** 2022-04-30

**Authors:** Félix Meléndez, Patricia Arroyo, Jaime Gómez-Suárez, Sergio Palomeque-Mangut, José Ignacio Suárez, Jesús Lozano

**Affiliations:** Industrial Engineering School, University of Extremadura, 06006 Badajoz, Spain; felixmv@unex.es (F.M.); parroyoz@unex.es (P.A.); jaimegs@unex.es (J.G.-S.); sergiopm@unex.es (S.P.-M.); jmarcelo@unex.es (J.I.S.)

**Keywords:** electronic nose, TCA, cork industry, machine olfaction, machine learning

## Abstract

2,4,6-trichloroanisole (TCA) is mainly responsible for cork taint in wine, which causes significant economic losses; therefore, the wine and cork industries demand an immediate, economic, noninvasive and on-the-spot solution. In this work, we present a novel prototype of an electronic nose (e-nose) using an array of digital and analog metal-oxide gas sensors with a total of 31 signals, capable of detecting TCA, and classifying cork samples with low TCA concentrations (≤15.1 ng/L). The results show that the device responds to low concentrations of TCA in laboratory conditions. It also differentiates among the inner and outer layers of cork bark (81.5% success) and distinguishes among six different samples of granulated cork (83.3% success). Finally, the device can predict the concentration of a new sample within a ±10% error margin.

## 1. Introduction

Artificial olfactory systems, commonly known as electronic noses (e-noses), are devices that try to mimic the biological sense of smell but eliminate its subjective component and exhaustion. The parallels between the two are enormous, as an artificial olfactory system reproduces all the stages of a biological one [1]. The first block of an e-nose is the sensing system, which consists of an array of gas sensors, each of which is sensitive in different ways to different aromas. The signals generated by these sensors pass to the learning stage, where the signals are first pre-processed and then pass through a pattern recognition system. Finally, these patterns are identified in the classification stage.

There are several types of gas sensors commonly used in electronic noses, such as electrochemical [2,3,4], digital Metal Oxide Semiconductor (MOS) [5,6], analog MOS [7,8,9,10], conducting polymer (CP) [11], surface acoustic wave (SAW) [12] and quartz crystal microbalances (QMB) sensors [13]. Some of these sensors, like digital MOS, have integrated their own microprocessor, analog/digital converter and a digital interface communication, such as I^2^C or SPI, all in a very small package of a few millimetres. This makes it easier for e-noses to become smaller and more portable.

E-noses have been used in a wide variety of applications and industries, with a current trend of using low-cost, low-consumption and low-size sensors. The application and the sensors used usually determine the portability of the e-nose. For example, devices have been developed to monitor urban pollution in fixed locations [14] and portable devices for monitoring indoor air quality [15]. Among other applications, they are also used in medicine to measure the air exhaled by patients for the early diagnosis of different diseases, such as lung cancer [16] or gastrointestinal disorders [17]. Electronic noses have even been used for exotic applications such as the detection of explosives [18] or drugs [19]. They have also gained popularity in determining the quality properties of food and drinks, where the applications are innumerable [20,21]. Within the field of beverages, special attention is paid to determining the quality of wine. In this respect, one of the materials most closely related to wine is the cork.

Cork is the material par excellence used to manufacture wine bottle stoppers, mainly due to its elastic, insulating, impermeable and durable properties [22]. However, natural cork can be contaminated with different substances produced by bacteria that, in contact with the wine, cause it to lose its organoleptic characteristics, leading to irreversible defects and consequent rejection by the consumer, as well as losses for wine producers. Some of these substances are 2,3,4,6-tetrachloroanisole (TeCA), 2,4,6-tribromoanisole (TBA) and 2-methylisoborneol (2-MIB), with 2,4,6-trichloroanisole (TCA) being the most common and important of all [23], and the target of this study.

As TCA is produced by bacteria, this substance can appear in localized points or areas of the cork planks, which means that contaminated and non-contaminated corks can come out of the same cork plank, making it difficult to detect and monitor the contaminant in the cork. Although there are non-destructive TCA detection techniques [24], one of the most widely used chemical analysis methods for its detection in the cork industry is gas chromatography [25,26,27]. This technique requires the destruction of the sample, considerable time to be carried out and it is conducted by specialised technicians. This means that in the industry, this technique is performed on random and representative samples of the batch produced, rather than on the whole batch, so that some TCA-contaminated closures may be put on the market. As an alternative to the use of these chemical analyses, the use of electronic noses is proposed. As an advantage, the e-nose can provide a non-destructive, faster and cheaper method of analysis compared to traditional methods.

Data processing of the signals obtained by an e-nose is of great importance to obtain an output that could be understood by non-technicians and to integrate e-noses into an online classification system. Among the most common techniques are Principal Component Analysis (PCA) [28], Support Vector Machines (SVM) [29], Artificial Neural Networks (ANN) [30] and Fuzzy Logic [31]. For this study, PCA and ANN techniques were used. PCA is a technique that reduces the dimensionality of a dataset into new uncorrelated variables (components) [32], which is quite useful for electronic noses due to the large number of sensors and, therefore, signals obtained. The ANN is a powerful classifier based on machine learning, consisting of interconnected layers of artificial neurons that are trained by adjusting the weight between neuron connections [33]. For the validation of the data obtained, the Leave One Out Cross-Validation (LOOCV) technique has been used, which is a procedure used to estimate the performance of machine learning algorithms when they are used to make predictions on data that are not used to train the model.

This article will describe the e-nose developed, its communication protocol with a smartphone, the measurements carried out, and the results and conclusions obtained. 

## 2. Materials and Methods

### 2.1. Description of the MultisensorNOSE

**Figure 1:**
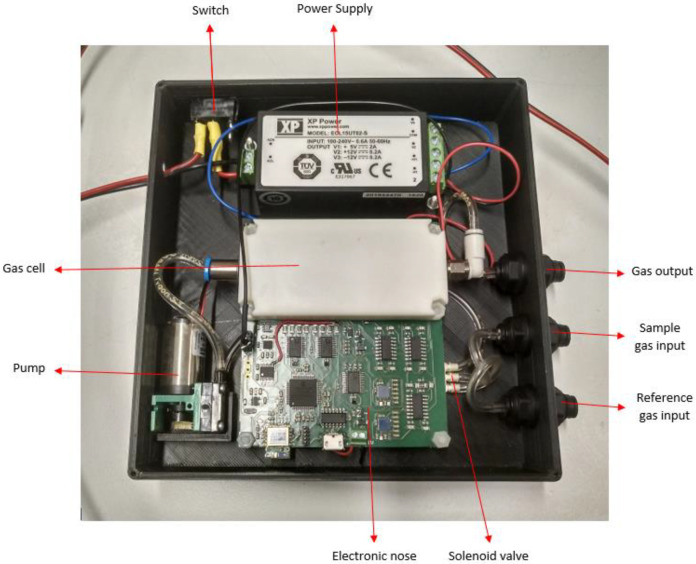
*MultisensorNOSE* top view.

The electronic nose, called *multisensorNOSE*, because of its number of sensors, is a home-designed and home-developed portable prototype with 14 sensors returning a total of 31 different signals. This device consists of a power supply that converts alternating current from 220 V_AC_ to 5 V_DC_, a 75 mm × 84.9 mm printed circuit board (PCB) that contains all the electronic circuitry of the e-nose, a pneumatic pump with fixed flow and a solenoid valve. All these components are housed inside a 3D printed case, which contains a switch to turn the device on-off and three-wall bushing that functions as inlets and outlets for the gases to be measured. The final size of the whole device is 150.76 mm × 147.40 mm × 35.20 mm, with a total weight of 450 g, which makes it a very easy and comfortable device to carry. The *multisensorNOSE* is shown in Figure 1.

**Figure 2:**
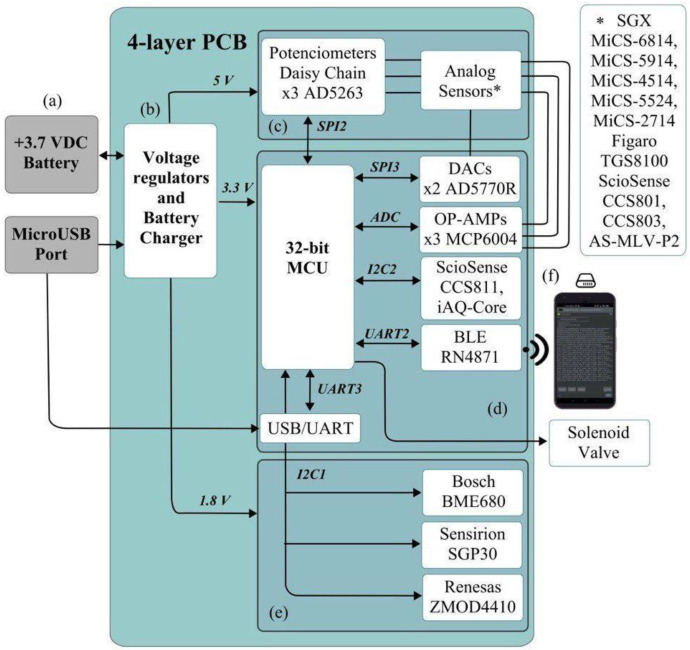
*MultisensorNOSE* block diagram. External battery (**a**); Voltage regulators and Battery Charger (**b**); 5 V_DC_ components (**c**); 3.3 V_DC_ components (**d**); 1.8 V_DC_ components (**e**); Smartphone connected via Bluetooth (**f**).

The PCB is represented in the block diagram in Figure 2. The e-nose is governed by a PIC32MM0256GPM064 microcontroller from Microchip Technology Inc. (Chandler, AZ, USA), which has 32 KB of data memory, 256 KB of programme memory, a frequency of 24 MHz, several I^2^C, SPI and UART modules, and an analog/digital converter of up to 24 channels and 12 bits of resolution, characteristics that make it an ideal microcontroller for this application. For external communication, a Bluetooth Low Energy module, Microchip’s RN4871, has been included, which allows communication via transparent UART.

As for the sensors, both digital and analog sensors were used (Table 1). Both types are based on MOS technology and have two types of resistors: the chemiresistive sensing element, which provides the signal, and the heater, whose function is to heat the sensor resistor to be able to operate. The digital sensors communicate via the I^2^C bus with the microcontroller and return the processed data in digital format. The signals from the analog resistors have to be amplified using some operational amplifiers, the Microchip’s MCP6004, and then converted to digital format in the microcontroller itself. Several AD5263 digital potentiometers from Analog Devices (Norwood, MA, USA) are included to vary the closed-loop gain of the operational amplifiers, communicating via I^2^C bus with the microcontroller. Moreover, two digital/analog current converters, Analog Devices’ AD5770R, have been used to inject current into the heaters of the analog sensors.

The e-nose circuitry also integrates voltage regulators that provide voltages of 1.8 V_DC_, 3.3 V_DC_ and 5 V_DC_, a connection to a solenoid valve controlled by the microcontroller, LEDs to indicate the status of the nose (on/off, measurements on/off) and a micro USB-B port connected to a battery charger, which allows using the device with an external 5 V power source. It can also communicate to an external device via a UART interface using the micro USB-B connector. 

Regarding the pneumatic connections, the housing has two gas inlets, one dedicated to clean or reference air and one for the sample to be sniffed. These two inlets communicate with the two inlets of the solenoid valve, whose output is connected to the pump inlet. The gas is directed towards the sensors from the pump outlet, which are encapsulated in a cell made by 3D resin printing. The output of this cell is connected directly to the output of the housing.

### 2.2. Communication Protocol

We developed an application for Android smartphones for data collection and configuration, using an ASCII code-based protocol for communication with the e-nose. In this way, we can quickly configure different parameters from the *multisensorNOSE*, such as adsorption time (reference air measurement time), desorption time (sample measurement time) and sampling period. In this application, there are four sections:

- Experiment: This section is where data are collected and stored with an easy user interface. Users can add smartphone GPS coordinates to the data and select the type of experiment. Buttons for starting and stopping the experiment and saving the data are available.
- Configuration: In this section, the user can change the adsorption and desorption times and the sampling period in an easy user interface.
- Graphics: In this section, the user can select one signal and see how it changes in real time using a graphic.
- Raw data: The application works like a UART terminal and shows all the data sent by the e-nose in this section.

**Figure 3:**
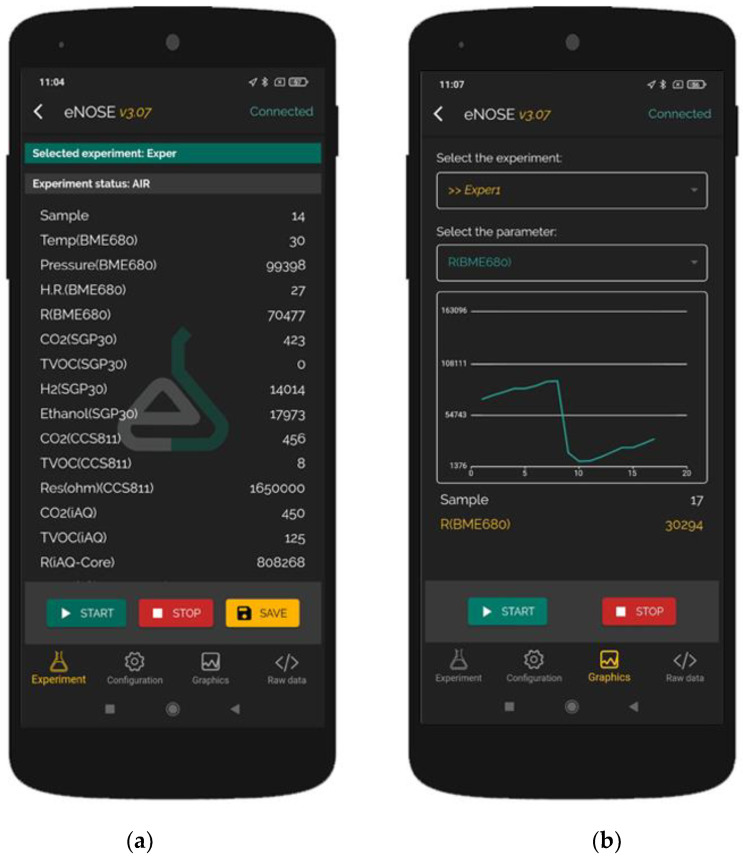
Developed application screenshots. (**a**) Data from the sensors; (**b**) Graphic with the time evolution from one of the signals.

The application, called eNoseLab, is shown in Figure 3.

The different types of experiments that the nose is capable of performing are shown in Table 2, which explains the name of the command, its ASCII code and a description. The experiment used for this study is one that contains all the sensors, *EXP_MAIN.*

### 2.3. Measurement Set-Up

To study the detection and discrimination capabilities of the device, several experiments have been carried out. Specifically, we measured TCA concentrations using permeation tubes in the lab and real cork samples (bark slab and granulated cork). With the first experiment, our goal is to assess whether the device can detect the compound at low concentrations. In the second, the aim is to test the ability to differentiate between the different layers of a cork slab. However, the third one was intended to evaluate the ability of the device to detect and quantify small changes in TCA concentration.

The first experiment consisted of detecting small concentrations of TCA in a gas flow, simulating real conditions to assess the performance of the device. To generate controlled gas properties mixed with water vapour, a gas generator and a humidity generator (models OVG-4 and OHG-4 from Owlstone, Westport, CT, USA) were used. Additionally, we used a commercial TCA permeation tube (KIN-TEK Analytical, Inc., La Marque, TX, USA) to produce a constant TCA concentration. Permeation tubes are plastic tubes with a semi-permeable membrane filled with a solid or liquid compound that releases this compound into the ambient air in gaseous form, depending on the temperature and the airflow. Then, knowing the characteristics of the compound and controlling the airflow and the temperature, it is possible to control the concentration of the compound that is being released.

**Figure 4:**
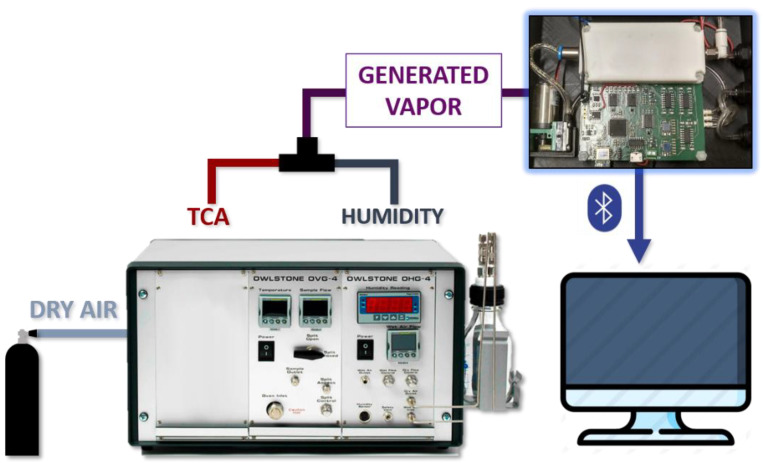
Experimental setup.

Figure 4 shows the experimental setup of the first experiment. The gas generator takes dry air from a bottle. This air flows through the cavity where the permeation tube is stored and through the humidity generator, thus producing two gas lines that are then mixed in a single line with known TCA concentration and humidity. This generated flows through the sensors and its responses are stored. The TCA permeation tube used was supplied by the manufacturer KIN-TEK Analytical (La Marque, TX, USA). This is a 10 cm tube with a permeation rate of 547 ng/min at 80 °C.

For the second experiment, samples from three different layers of tree bark were measured. The TCA usually concentrates in the outer zone of the bark. The sample of layer 1 corresponds to the outer zone, layer 2 corresponds to the intermediate zone, and layer 3 corresponds to the inner zone of the bark. To carry out the measurements, 2 pieces of 4 grams (±0.5) were cut for each layer. These samples were placed in glass vials, and then the static headspace was transferred by active sampling with a pump to the gas cell (flow rate approximately 170 mL/m). Eighteen measurement cycles were performed for each sample. Each measurement cycle (2 min) is composed of two phases with a 1-min duration: the filtered air phase and the sample phase. In this way, the solenoid valve switches the air inlet between filtered air (used as a reference) and the corresponding sample headspace.

**Figure 5:**
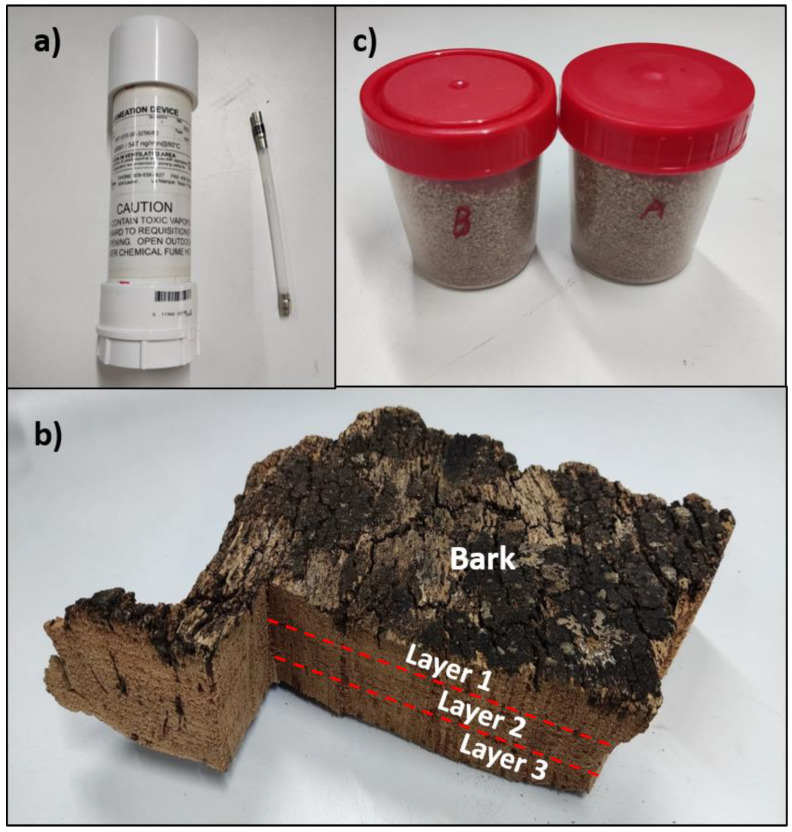
Samples. (**a**) Permeation tube with TCA; (**b**) Layers of bark slab; (**c**) Granulated cork samples.

In the last experiment, granulated cork samples were measured at 6 different TCA concentrations: 4.1 ng/L, 6.5 ng/L, 8.3 ng/L, 10.7 ng/L, 12.4 ng/L and 15.1 ng/L. These samples were provided by a cork stopper factory, DIAM Corchos S.A. (Badajoz, Spain), and the concentrations were calculated following the UNE 56930:2017 standard. The samples will be named A, B, C, D, E and F, respectively, in the results section. For this test, 2 g of a sample were placed in the vials. The rest of the measurement protocol was the same as the one described above for the experiment with cork slabs. A picture of the samples that were used in experiments one, two and three are shown in Figure 5.

## 3. Results and Discussion

### 3.1. Gas Generator

**Figure 6:**
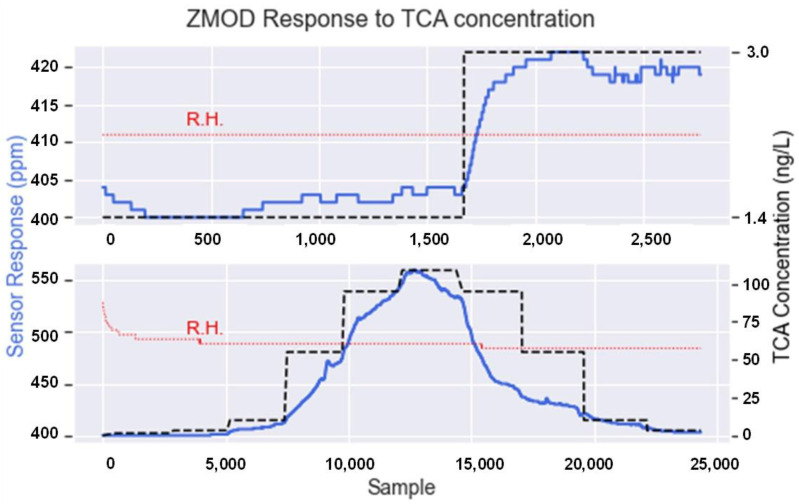
ZMOD4410 response to different TCA concentrations. The black dashed line represents the TCA concentration, the blue line represents the sensor response, and the red dotted lines show that the relative humidity remained constant during the experiment (33% R.H. approx.).

With the previous setup, two different approaches were considered. First, a single concentration step was generated (Figure 6, top). In this case, the TCA concentration ranged from 1.4 ng/L, which is the minimum that the system could generate, to 3.0 ng/L. This first step allowed us to confirm that the e-nose detected small concentration variations. Second, we presented the sensors to a wider range of TCA concentrations (Figure 6 bottom), from 1.4 to 110 ng/L, to confirm the recovery of the sensors after being exposed to high concentrations. During this process, relative humidity was set to a constant value so we could assess that the changes in the sensors’ response were only due to variations in TCA concentration and not by the effect of humidity.

### 3.2. Cork Slab

The concentration of TCA is known to be higher in the outermost part of the tree bark [34]. Therefore, first, a test was carried out to study whether the system can differentiate between different layers of the tree bark. Once all the measurements were obtained, baseline manipulation pre-processing was performed following Equation (1).

**Figure 7:**
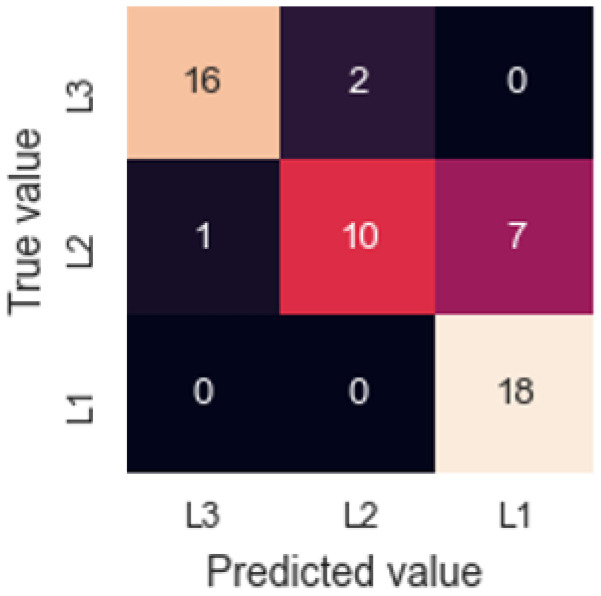
Confusion matrix for cork slab results.

Characteristic Value = (Reference/Sample) · 100,(1)

where Characteristic Value is the pre-processed value, Reference is an average of the last 5 values reached during the clean air measurement phase and Sample is an average of the last 5 values reached during the analyte measurement phase. A neural network was then trained using Rectified Linear Unit activation function and 4 hidden layers. Using a Leave One Out Cross-Validation (LOOCV), a success rate of 81.5% is achieved. Figure 7 shows the resulting confusion matrix, where it is observed that 7 of the errors are made in the classification of samples from layer 2 as layer 1. This interference may be due to the similarity in the TCA concentration between these two outermost layers. However, the device can differentiate very successfully between the innermost layer (L3) and the outermost layer (L1).

### 3.3. Granulated Cork

**Figure 8:**
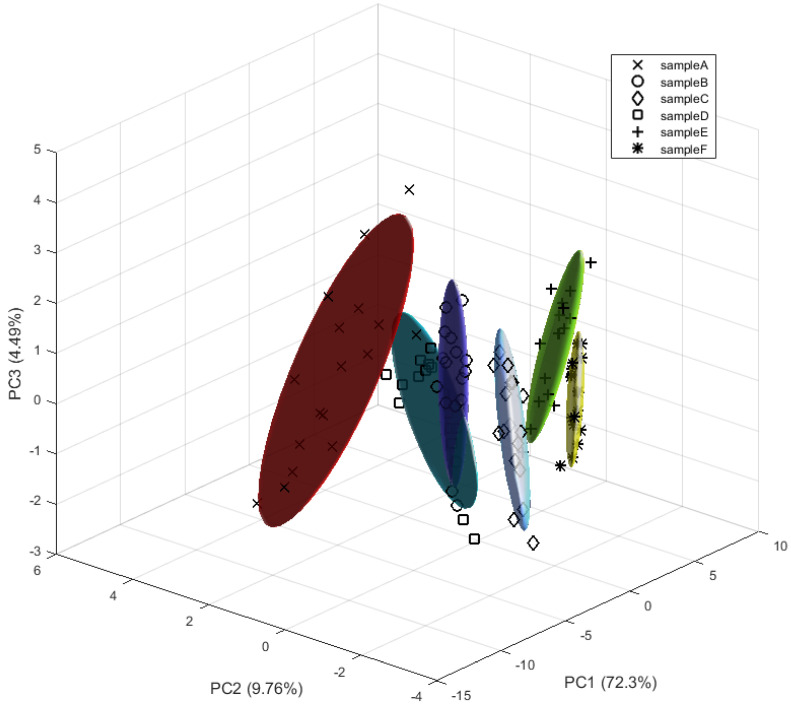
PCA plot for measurements with granulated corks at different concentrations.

The procedure followed for the pre-processing of these data is the same as described in the previous section. In this case, the first repetition or cycle of each sample was discarded. Principal component analysis (PCA) of the pre-processed data was then performed. By selecting the first three components, 86.6% of the variance in the data was explained. Short-term drift through PC3 can be seen in Figure 8. This effect was attributed to the experimental procedure; the recipient was opened and cleaned after each measurement. Long-term drift was not considered given the length of our experiments, but it should be addressed in future works.

Figure 8 shows that the clusters can be broadly differentiated, but there is an overlap between samples B and D.

**Figure 9:**
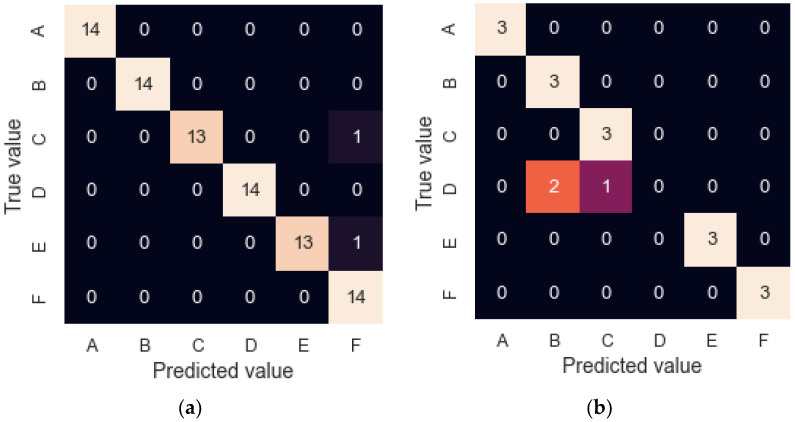
Confusion matrices for granulated cork results. (**a**) LOOCV with 14 cycles; (**b**) CV with the remaining three cycles.

In addition, to study the classification capacity, a neural network with the same programming characteristics was trained. However, in this case, it was trained and validated (LOOCV) with 14 repetitions per sample and re-validated with the remaining three. The model achieved a 94.0% success rate in the model validation step and an 83.3% rate when classifying three new repetitions. The confusion matrices obtained in both cases are shown in Figure 9. Once the new three samples were presented, the model successfully classified all of them but sample C, which was classified as sample B and C, meaning the model underestimated the concentration of sample C, which could be caused by the ageing or heterogeneity of the samples.

**Figure 10:**
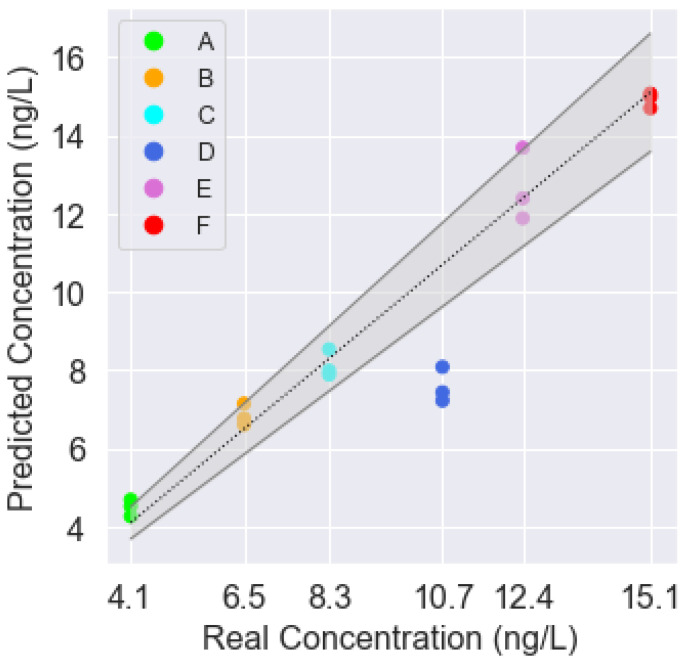
Results of the prediction model.

Since the classification success rate was adequate, we decided to programme a predictor to determine if it was possible to predict the estimated concentration of TCA present in the sample. The programmed features were the same as for the classifier. The results can be seen in Table 3 and Figure 10, where the predicted vs. actual concentration values are plotted. The grey dotted line represents the 1:1 line, whereas the grey solid lines represent a ±10% error margin of the real concentration. 

It was observed that, once again, errors appeared with sample D since the system detects a lower TCA concentration than the expected one (23–25% lower). For every other sample, the predicted concentration was very close to or within the ±10% error margin. The concentration of sample A was 12-2% higher. For sample B, the concentration was 9-2% above the real concentration. In the case of sample C, the model predicted a TCA concentration 2% higher or 6% lower than the real concentration. The concentration of sample E was within the range 95–109%. Finally, sample F had the lowest deviation from the real concentration, only 1–3% lower. If all 18 samples were considered, the performance of the device could be estimated with error rates of R^2^ = 0.87, MAE = 0.84 ng/L and RMSE = 1.36 ng/L.

## 4. Conclusions

In this work, we designed and built an electronic olfaction system based on an array of 14 digital and analog sensors (31 signals) with the aim of detecting 2,4,6-trichloroanisole in cork. Three different experiments were conducted. First, generating known TCA concentrations in a gas generator; second, using a bark slab sample; and third, using granulated cork.

We confirmed that the device detects low variations in the concentration of the compound (1.4–3.0 ng/L). The performance of the e-nose when higher concentrations (up to 110 ng/L) were presented was also evaluated.

The e-nose differentiated between the different layers of the bark slab with an 81.5% success rate, thus detecting those parts with higher TCA concentrations.

With the use of granulated cork, six different samples (A–F) with TCA concentrations ranging from 4.1 to 15.1 ng/L were presented to the device. The principal component analysis showed that the device could identify the samples. To quantify this, we used a classifier neural network model, which, after training, achieved an 83.3% success rate. A prediction model was also built demonstrating that the system could predict the TCA concentration within a ±10% concentration range (R^2^ = 0.87, MAE = 0.84 and RMSE = 1.36).

The overall performance of the device was satisfactory. The work presented in this paper is intended to be a further step in our goal of detecting TCA, helping the cork industry to develop an online, immediate and low-cost system for detecting faulty cork stoppers. Future work will focus on improving the design of the device and carrying out experiments with real cork stoppers to differentiate clean stoppers from those with high TCA concentrations. In addition, a comparison of sensitivity and accuracy will be made with other TCA detection techniques, such as spectroscopy and gas chromatography.

## Figures and Tables

**Table 1:** Sensors used in *multisensorNOSE* and output signals.

Sensor Model	Manufacturer	Type	Output Signals
BME680	Bosch Sensortech GmbH, Germany	Digital	Temperature, Relative Humidity, Pressure, Resistance Value
CCS811	ScioSense B.V., The Netherlands	Digital	CO_2_, TVOCs ^1^, Resistance Value
SGP30	Sensirion AG, Switzerland	Digital	CO_2_, TVOCs, H_2_ (raw signal ^2^), Ethanol (raw signal)
iAQ-Core C	ScioSense B.V., The Netherlands	Digital	CO_2_, TVOCs, Resistance Value
ZMOD4410	Renesas Electronics Corporation, Japan	Digital	Ethanol (raw signal), Resistance Value, CO_2_, TVOC, IAQ ^3^
MiCS-2714	SGX Sensortech, Switzerland	Analog	NO_2_
MiCS-5524	SGX Sensortech, Switzerland	Analog	CO
MiCS-4514	SGX Sensortech, Switzerland	Analog	CO, NO_2_
MiCS-5914	SGX Sensortech, Switzerland	Analog	NH_3_
MiCS-6814	SGX Sensortech, Switzerland	Analog	CO, NO_2_, NH_3_
CCS801	ScioSense B.V., The Netherlands	Analog	VOCs
CCS803	ScioSense B.V., The Netherlands	Analog	Ethanol
TGS8100	Figaro Engineering Inc., Japan	Analog	VOCs
AS-MLV-P2	ScioSense B.V., The Netherlands	Analog	VOCs

^1^ Total Volatile Organic Compounds. ^2^ Pre-processed signal from sensor resistance. ^3^ Air Quality Index.

**Table 2:** ASCII codes for *multisensorNOSE* experiments commands.

Command	ASCII Code	Description
MEAS_BME	BME680\r\n	Send a BME680 measure
MEAS_SGP	SGP30\r\n	Send a SGP30 measure
MEAS_CCS	CCS811\r\n	Send a CCS811 measure
MEAS_IAQ	iAQ-Core\r\n	Send an iAQ-Core measure
MEAS_ZM	ZMOD4410\r\n	Send a ZMOD4410 measure
MEAS_SAN	SenAn\r\n	Send from all analog sensors
EXP_MAIN	Exper\r\n	Initiate main experiment
EXP_BME	Exper1\r\n	Initiate only BME680 experiment
EXP_SGP	Exper2\r\n	Initiate only SGP30 experiment
EXP_CCS	Exper3\r\n	Initiate only CCS811 experiment
EXP_IAQ	Exper4\r\n	Initiate only iAQ-Core experiment
EXP_ZM	Exper5\r\n	Initiate only ZMOD4410 experiment
EXP_SAN	Exper6\r\n	Initiate only analog sensors experiment
STOP	Stop\r\n	Stop experiment
INFO	INFO\r\n	Send device details

**Table 3:** Real concentration and predicted concentration in ng/L.

Class	Real Concentration (ng/L)	Predicted Concentration (ng/L)
A	4.1	4.5
A	4.1	4.6
A	4.1	4.2
B	6.5	6.7
B	6.5	7.1
B	6.5	6.6
C	8.3	8.5
C	8.3	7.8
C	8.3	7.9
D	10.7	7.2
D	10.7	7.4
D	10.7	8.0
E	12.4	11.8
E	12.4	12.3
E	12.4	13.6
F	15.1	14.9
F	15.1	15.0
F	15.1	14.6

## Data Availability

Not applicable.
